# Supplementary material for: Mutations in the PIK3C2B, ERBB3, KIT, and MLH1 Genes and Their Relationship with Resistance to Temozolomide in Patients with High-Grade Gliomas
Source: Biomedicines. 2024 Dec 6;12(12):2777. doi: 10.3390/biomedicines12122777 (PMC11673431; doi:10.3390/biomedicines12122777)
Supplement: Supplementary file 1 [file biomedicines-12-02777-s001.zip › biomedicines-3205381-supplementary.pdf]

Supplementary Material 1

Mutations in the *PIK3C2B*, *ERBB3*, *KIT*, and *MLH1* Genes and their Relationship with Resistance to Temozolomide in Patients with High-grade Gliomas

León Darío Ortiz Gómez [1, 2], Heidy Johanna Contreras Martínez [3], David Andrés Galvis Pareja [3], Sara Vélez Gómez[4], Jorge Emilio Salazar Flórez [5], Fernando P. Monroy [6] and Ronald Guillermo Peláez Sánchez [4, *]

Correspondence:
Ronald Guillermo Peláez Sánchez, rpelaezp@ces.edu.co, Phone: (57) (604) 4440555 Extension 1750

Supplementary Material 1. Summary table 1, the table shows the patient's identification number, clinical diagnosis, tumor classification by conventional tests (codeletion 1p/19q, Mutation IDH, MGMT promoter methylation), the type of sample used for genomic profiling, the mutations found after genomic profiling (useful for reclassifying patients), and finally the survival time (progression-free and overall survival). (-) Patients without data.

**Patient number**	**Glioma entity**	**Codeletion 1p/19q (FISH)**	**IDH mutation (IHQ)**	**MGMT promoter methylation (PCR)**	**Sample type (F-1CDX)**	**Mutations in cancer-related genes**	**Mutations of uncertain significance**	**Free progression first relapse (months)**	**Free progression second relapse (months)**	**Overall survival (months)**
1	Glioblastoma	-	wild type	unmethylated	Paraffin block	*BCORL1, CDH1, HGF, TERT*	*MAP3K13, SPEN, MED12, TSC1, RAD51D, RICTOR*	13.6	17.6	19.4.
2	Glioblastoma	-	-	-	Paraffin block	*EGFR, PIK3CA, CDKN2AB, NOTCH1, TERT, TP53*	*ALK, MYD88, TEK, ALOX12B, NOTCH1, CD79A, POLE, MPL, RET*	18.3	34.3	38
3	Glioblastoma	-	wild type	unmethylated	Liquid biopsy		*EP300, NOTCH3, ERBB3, MSH2, MSH3*	10.1	13.1	17.1
4	Glioblastoma	-	wild type	unmethylated	Paraffin block	*KIT, PDGFRA, KDR, CDKN2A/B, TERT*	*AR, TSC1, DIS3, PPP2RIA, SNCAIP*	7.9	13.6	18
5	Glioblastoma	-	-	-	Paraffin block	*EGFR, PTEN, CDKN2A/B, MTAP, TERT*	*ARAF, RPTOR, ESR1, LTK, MUTYH*	23.4	37.2	42

Patient number	Glioma entity	Codeletion 1p/19q (FISH)	IDH mutation (IHQ)	MGMT promoter methylation (PCR)	Sample type (F-1CDX)	Mutations in cancer-related genes	Mutations of uncertain significance	Free progression first relapse (months)	Free progression second relapse (months)	Overall survival (months)
6	Glioblastoma	-	wild type	-	Paraffin block	*EGFR, PTEN, CDK2NA/B, MTAP, TERT*	*EGFR, RPTOR, ERBB2, TEK, FGFR1, FLT1*	7.9	19.7	27.1
7	Glioblastoma	-	wild type	unmethylated	Paraffin block	*EGFR, KIT, PDGFRA, CDK4, KEAP1, MDM2, PIK3R1, TERT*	*CCN2, PPARG, MAF, TET2, NOTCH3, PIK3C2G*	14.8	47.2	54
8	Glioblastoma	-	-	-	Paraffin block	*CDK4, FGFR3, MDM2, TERT*	*ERBB3, RPTOR, JAK2, KMT2A(MLL), MDM2*	9.8	15.1	18.8
9	Glioblastoma	-	-	unmethylated	Liquid biopsy	*ATM, DNMT3A*	*POLE, RPTOR, SPEN, TSC1*	11	19.1	24
10	Glioblastoma	-	wild type	-	Liquid biopsy	*FANCL, NRAS, MSH6*	*GNAS, VHL, KIT, MED12, MLH1*	3		6.4
11	Glioblastoma	-	-	unmethylated	Paraffin block	*NF1-CDK6, HGF, CBL, PTPN11, TERT*	*ALK, NF2, GRM3, PIK3RI, IRF4, TSC1, MML2*	10.3	14.2	live
12	Glioblastoma	-	-	-	Paraffin block	*PDGFRA, CCND2, FGF23, FGF6, KDM5A, NTRK1, TP53*	*BRIP1, MAF, SMAD2, BTK, NOTCH2, SPEN, CDHH1, RAD52,*	8	13.1	20

Patient number	Glioma entity	Codeletion 1p/19q (FISH)	IDH mutation (IHQ)	MGMT promoter methylation (PCR)	Sample type (F-1CDX)	Mutations in cancer-related genes	Mutations of uncertain significance	Free progression first relapse (months)	Free progression second relapse (months)	Overall survival (months)
							TYR03, KEAP1, RET			
13	Glioblastoma	-	mutated	methylated	Paraffin block	*NF1, CDKN2A/B, CDKN2C, MTAP, MUTYH, TOTN11, SETD2, TERT, TP53*	*BTB2, RAD54L, CBL, CIC, FANCA*	14.1	21.2	live
14	Glioblastoma	-	-	-	Paraffin block	*BRAF, CDKN2A/B, TERT*	*ERBB3, ZNF703, NOTCH1, PDCD1LG(PD-L2), SGK1*	13.3	23.6	28.7
15	Glioblastoma	-	-	-	Paraffin block	*MDM4, PIK3C2B, TERT*	*ABL, PIK3C2B rearrangement, AXIN1, PTEN, EED, MSH2*	2.2	2.5	9.7
16	Glioblastoma	-	mutated	methylated	Paraffin block	*NF1, ATRX, MLH1, RB1, TP53*	*ABL1, KEL, SPEN, CREBBP, MEF2B, EP300, NOTCH3, INPP4B, PMS2*	16.1	79.3	84.5
17	Astrocytoma	-	Mutated	methylated	Paraffin block	*IDH1, TP53*	*ATRX, MED12, CD22, ZNF703, EP300, KDR*	23.1	26.6	34

Patient number	Glioma entity	Codeletion 1p/19q (FISH)	IDH mutation (IHQ)	MGMT promoter methylation (PCR)	Sample type (F-1CDX)	Mutations in cancer-related genes	Mutations of uncertain significance	Free progression first relapse (months)	Free progression second relapse (months)	Overall survival (months)
18	Glioblastoma	-	mutated	unmethylated	Paraffin block	*EGFR, ARID1A, RB1, TERT, TP53*	*AR, MLH1, EGFR, PRKCI, EP300, RET, ESR1, TP53*	6.6	10.1	15.4
19	Astrocytoma	-	-	-	Paraffin block	*IDH1, ATRX, KEL, TP53*	*GATA4, TYR03*	18.4	28.8	live
20	Glioblastoma	-	-	-	Paraffin block	*STAT3, RB1, STAG2, TERT, TP53*	*BCOR, U2AF1, FLT1, HSD3D, NOTCH2*	21.1	32.4	36.6
21	Glioblastoma	-	-	-	Paraffin block	*BCOR, MUTYH, U2AF1*	*AXL, KDR, SET2, BRD4, KEL, SOX9, BTK, MRE11A, DNMT3A, PTCH1*	12.5	26.2	31
22	Astrocytoma	-	mutated	methylated	Paraffin block	*CDKN2A/B, IDH2, MTAP, NOTCH1, TERT*	*ATR, DIS3, NTRK1, BRCA1, FANCC, PIK3R1, BRCA2, GNAS, PRDM1, BRIP1, NOTCH1, SMARCA4*	61.4	73	87.7
23	Astrocytoma	-	mutated	-	Liquid biopsy	*TP53*	*AXIN1, TSC1, BRCA2, MAP2K2 (MEK2), MAP3K1*	6.8	11.7	live

Patient number	Glioma entity	Codeletion 1p/19q (FISH)	IDH mutation (IHQ)	MGMT promoter methylation (PCR)	Sample type (F-1CDX)	Mutations in cancer-related genes	Mutations of uncertain significance	Free progression first relapse (months)	Free progression second relapse (months)	Overall survival (months)
24	Glioblastoma	-	mutated	methylated	Paraffin block	*NF1, CARD11, SETD2, STAG2, TP53*	*ALK, MLL2, ASXL1, NOTCH3, BTK, PIK3C2B, EPHB4*	8.6	13	14,5
25	Glioblastoma	-	-	unmethylated	Paraffin block	*EP300, SETD2*	*NTRK1, TET2, RAD54L, TSC1, SPEN, TBX3*	10.1	13.3	19
26	Oligodendroglioma	1p/19q+	mutated	-	Paraffin block	*IDH1, SOX2, CIC, TERT*	*CXCR4, TEK, FGF19, TGFBR2, IRS2, Soen*	52.6	64.4	live
27	Oligodendroglioma	1p/19q+	mutated	methylated	Paraffin block	*IDH1, NF1, TP53*	*CARD11, NTRK2, FGF6, SMARCA4, GABRA6, MSH2*	238.4	253.9	live
28	Glioblastoma	-	mutated	-	Liquid biopsy	*ABL1*	*ATR, PPP2R1A, BCL2, SDHA, NF1, TSC1, NOTCH2, ZNF703*	15.2	20.4	live
29	Oligodendroglioma	1p/19q+	mutated	methylated	Paraffin block	*IDH1, ARID1A, CIC, TERT*	*ALK, DIS3, MLL2, TSC2, AXL, FLCN, POLE, BRD4, FLT3, ROS1, CREBBP, LTK, SPEN*	145.7	152.7	live

Patient number	Glioma entity	Codeletion 1p/19q (FISH)	IDH mutation (IHQ)	MGMT promoter methylation (PCR)	Sample type (F-1CDX)	Mutations in cancer-related genes	Mutations of uncertain significance	Free progression first relapse (months)	Free progression second relapse (months)	Overall survival (months)
30	Astrocytoma	-	mutated	methylated	Paraffin block	*IDH1, CDH1, TP53*	*ATRX, KLHL6, DIS3, MLL2, FANCL, MSH3, KDR, POLD1.*	10.1	13.3	45
31	Oligodendroglioma	1p/19q	mutated	methylated	Paraffin block	*IDH2, ARIDIA, CIC, FUBP1, NOTCH1, TERT, TP53*	*BCOR, MAP3K1, TSC1, CIC, POLE, FGFR1, NFR43, KIT, ROS1.*	13.6	24.9	32.1

Supplementary Material 1. Summary table 2. Genes with mutations, the table shows the genes with some type of mutation that were found in the 31 patients with high-grade gliomas, classified according to the type of high-grade glioma.

Gene	Glioblastoma (GB)				Astrocytoma				Oligodendroglioma			
	Not		yes		Not		yes		Not		yes	
	n	%	n	%	n	%	n	%	n	%	n	%
ABL1	19	86	**3**	14	5	100	0	0	4	100	0	0
AKT1	22	100	0	0	5	100	0	0	4	100	0	0
AKT2	22	100	0	0	5	100	0	0	4	100	0	0
**ALK**	19	86	3	14	5	100	**0**	0	3	75	1	25
ALOX12B	20	91	**2**	9	5	100	0	0	4	100	0	0
APC	22	100	0	0	5	100	0	0	4	100	0	0
AR	20	91	**2**	9	5	100	0	0	4	100	0	0
ARAF	21	96	**1**	4	5	100	0	0	4	100	0	0
**ARID1A**	21	96	1	4	5	100	0	0	2	50	2	50
ASXL1	21	96	1	4	5	100	0	0	4	100	0	0
ATM	21	96	**1**	4	5	100	**0**	0	4	100	0	0
ATR	22	100	**0**	0	4	80	1	20	4	100	0	0
ATRX	22	100	0	0	2	40	**3**	60	4	100	0	0
AURKB	22	100	0	0	5	100	**0**	0	4	100	0	0
AXIN1	21	96	1	4	4	80	**1**	20	4	100	0	0
AXL	21	96	1	4	5	100	0	0	3	75	1	25
BAP1	22	100	**0**	0	5	100	0	0	4	100	0	0
BCL2	21	96	**1**	4	5	100	0	0	4	100	0	0
**BCOR**	20	91	2	9	5	100	0	0	3	75	1	25
BCORL1	22	100	**0**	0	5	100	**0**	0	4	100	0	0
BRAF	21	96	1	4	5	100	0	0	4	100	0	0
BRCA1	22	100	0	0	4	80	1	20	4	100	0	0
BRCA2	22	100	0	0	3	60	2	40	4	100	0	0
BRD4	21	96	**1**	4	5	100	0	0	3	75	1	25

BRIP1	21	96	**1**	4	4	80	**1**	20	4	100	**0**	0
BTB2	21	96	**1**	4	5	100	**0**	0	4	100	0	0
BTK	19	86	**3**	14	5	100	**0**	0	4	100	0	0
C11ORF30EMSY	22	100	**0**	0	5	100	0	0	4	100	**0**	0
CARD11	21	96	1	4	5	100	**0**	0	3	75	1	25
CASP8	22	100	0	0	5	100	0	0	4	100	0	0
CBL	20	91	2	9	5	100	0	0	4	100	0	0
**CCND2**	20	91	2	9	5	100	0	0	4	100	0	0
CD22	22	100	0	0	4	80	1	20	4	100	0	0
CD274PDL1	22	100	0	0	5	100	0	0	4	100	0	0
CD79A	20	91	2	9	5	100	0	0	4	100	0	0
CDH1	21	96	1	4	4	80	1	20	4	100	0	0
CDK4	20	91	2	9	5	100	0	0	4	100	0	0
CDK6	21	96	1	4	5	100	0	0	4	100	0	0
**CDKN2AB**	17	77	5	23	5	100	0	0	4	100	0	0
CDKN2C	21	96	1	4	5	100	0	0	4	100	0	0
**CIC**	21	96	1	4	5	100	0	0	0	0	4	100
CREBBP	21	96	1	4	5	100	0	0	3	75	1	25
CSF1R	22	100	0	0	5	100	0	0	4	100	0	0
CXCR4	22	100	0	0	5	100	0	0	3	75	1	25
CDHH1	21	96	1	4	5	100	0	0	4	100	0	0
DDR1	22	100	0	0	5	100	0	0	4	100	0	0
DIS3	21	96	1	4	3	60	2	40	3	75	1	25
DNMT3A	20	91	2	9	5	100	0	0	4	100	0	0
DOT1L	22	100	0	0	5	100	0	0	4	100	0	0
EED	21	96	1	4	5	100	0	0	4	100	0	0
**EGFR**	15	68	7	32	5	100	0	0	4	100	0	0

**EP300**	18	82	4	18	4	80	1	20	4	100	0	0
EPHA3	22	100	0	0	5	100	0	0	4	100	0	0
EPHB1	22	100	0	0	5	100	0	0	4	100	0	0
EPHB4	21	96	1	4	5	100	0	0	4	100	0	0
ERBB2	21	96	1	4	5	100	0	0	4	100	0	0
ERBB3	19	86	3	14	5	100	0	0	4	100	0	0
ESR1	21	96	1	4	5	100	0	0	4	100	0	0
FAM123B	22	100	0	0	5	100	0	0	4	100	0	0
FANCA	21	96	1	4	5	100	0	0	4	100	0	0
FANCC	22	100	0	0	4	80	1	20	4	100	0	0
FANCG	22	100	0	0	5	100	0	0	4	100	0	0
FANCL	21	96	1	4	4	80	1	20	4	100	0	0
FBXW7	22	100	0	0	5	100	0	0	4	100	0	0
FGF19	22	100	0	0	5	100	0	0	3	75	1	25
FGF23	21	96	1	4	5	100	0	0	4	100	0	0
FGF6	21	96	1	4	5	100	0	0	3	75	1	25
**FGFR1**	21	96	1	4	5	100	0	0	3	75	1	25
FGFR3	21	96	1	4	5	100	0	0	4	100	0	0
FGFR4	22	100	0	0	5	100	0	0	4	100	0	0
FLCN	22	100	0	0	5	100	0	0	3	75	1	25
FLT1	20	91	2	9	5	100	0	0	4	100	0	0
FLT3	22	100	0	0	5	100	0	0	3	75	1	25
**FUBP1**	22	100	0	0	5	100	0	0	3	75	1	25
GABRA6	22	100	0	0	5	100	0	0	3	75	1	25
GATA4	22	100	0	0	4	80	1	20	4	100	0	0
GNAS	21	96	1	4	4	80	1	20	4	100	0	0
GRM3	21	96	1	4	5	100	0	0	4	100	0	0
GSK3B	22	100	0	0	5	100	0	0	4	100	0	0

HGF	20	91	2	9	5	100	0	0	4	100	0	0
HSD3B1	22	100	0	0	5	100	0	0	4	100	0	0
ID3	22	100	0	0	5	100	0	0	4	100	0	0
**IDH1**	22	100	0	0	2	40	3	60	1	25	3	75
**IDH2**	22	100	0	0	4	80	1	20	3	75	1	25
IKZF1	22	100	0	0	5	100	0	0	4	100	0	0
INPP4B	21	96	1	4	5	100	0	0	4	100	0	0
IRF4	21	96	1	4	5	100	0	0	4	100	0	0
IRS2	22	100	0	0	5	100	0	0	3	75	1	25
JAK2	21	96	1	4	5	100	0	0	4	100	0	0
JAK3	22	100	0	0	5	100	0	0	4	100	0	0
KDM5A	21	96	1	4	5	100	0	0	4	100	0	0
**KDR**	20	91	2	9	3	60	2	40	4	100	0	0
**KEAP1**	21	96	1	4	5	100	0	0	4	100	0	0
KEL	20	91	2	9	4	80	1	20	4	100	0	0
KIT	19	86	3	14	5	100	0	0	3	75	1	25
KLHL6	22	100	0	0	4	80	1	20	4	100	0	0
KMT2AMLL	21	96	1	4	5	100	0	0	4	100	0	0
KRAS	22	100	0	0	5	100	0	0	4	100	0	0
LTK	21	96	1	4	5	100	0	0	3	75	1	25
**MAF**	20	91	2	9	5	100	0	0	4	100	0	0
MAP2K1MEK1	22	100	0	0	5	100	0	0	4	100	0	0
MAP2K2MEK2	22	100	0	0	4	80	1	20	4	100	0	0
**MAP3K1**	22	100	0	0	4	80	1	20	3	75	1	25
MAP3K13	21	96	1	4	5	100	1	20	4	100	0	0
MDM2	19	86	3	14	5	100	0	0	4	100	0	0
MDM4	21	96	1	4	5	100	0	0	4	100	0	0
MED12	21	96	1	4	4	80	1	20	4	100	0	0

MEF2B	21	96	1	4	5	100	0	0	4	100	0	0
MET	22	100	0	0	5	100	0	0	4	100	0	0
MLH1	19	86	3	14	5	100	0	0	4	100	0	0
**MLL2**	21	96	1	4	4	80	1	20	4	100	0	0
MPL	21	96	1	4	5	100	0	0	4	100	0	0
MRE11A	21	96	1	4	5	100	0	0	4	100	0	0
MSH2	20	91	2	9	5	100	0	0	3	75	1	25
MSH3	21	96	1	4	4	80	1	20	4	100	0	0
MSH6	21	96	1	4	5	100	0	0	4	100	0	0
MTAP	20	91	2	9	5	100	0	0	4	100	0	0
MUTYH	19	86	3	14	5	100	0	0	4	100	0	0
MYD88	21	96	1	4	5	100	0	0	4	100	0	0
MTAP	21	96	1	4	4	80	1	20	4	100	0	0
**NF1**	17	77	5	23	5	100	0	0	3	75	1	25
NF2	21	96	1	4	5	100	0	0	4	100	0	0
NFR43	22	100	0	0	5	100	0	0	3	75	1	25
**NOTCH1**	18	81	4	19	4	80	1	20	3	75	1	25
NOTCH2	19	86	3	14	5	100	0	0	4	100	0	0
NOTCH3	18	82	4	18	5	100	0	0	4	100	0	0
NRAS	21	95	1	5	5	100	0	0	4	100	0	0
NTRK1	20	91	2	9	4	80	1	20	4	100	0	0
NTRK2	22	100	0	0	5	100	0	0	3	75	1	25
PARP2	22	100	0	0	5	100	0	0	4	100	0	0
PAX5	22	100	0	0	5	100	0	0	4	100	0	0
PBRM1	22	100	0	0	5	100	0	0	4	100	0	0
PDCD1LG2PDL2	21	95	1	5	5	100	0	0	4	100	0	0
**PDGFRA**	19	86	3	14	5	100	0	0	4	100	0	0
PIK3C2B	19	86	3	14	5	100	0	0	4	100	0	0

PIK3C2G	21	95	1	5	5	100	0	0	4	100	0	0
PIK3CA	21	95	1	5	5	100	0	0	4	100	0	0
PIK3R1	20	91	2	9	4	80	1	20	4	100	0	0
PMS2	21	95	1	5	5	100	0	0	4	100	0	0
POLD1	22	100	0	0	4	80	1	20	4	100	0	0
**POLE**	20	91	2	9	5	100	0	0	2	50	2	50
PPARG	21	95	1	5	5	100	0	0	4	100	0	0
PPP2R1A	20	91	2	9	5	100	0	0	4	100	0	0
PRKCI	21	95	1	5	5	100	0	0	4	100	0	0
PTCH1	21	95	1	5	5	100	0	0	4	100	0	0
PTEN	19	86	3	14	5	100	0	0	4	100	0	0
PTPN11	21	95	1	5	5	100	0	0	4	100	0	0
PTPRO	22	100	0	0	5	100	0	0	4	100	0	0
QKI	22	100	0	0	5	100	0	0	4	100	0	0
RAC1	22	100	0	0	5	100	0	0	4	100	0	0
RAD21	22	100	0	0	5	100	0	0	4	100	0	0
RAD51D	21	95	1	5	5	100	0	0	4	100	0	0
RAD52	21	95	1	5	5	100	0	0	4	100	0	0
RAD54L	20	91	2	9	5	100	0	0	4	100	0	0
RB1	19	86	3	14	5	100	0	0	4	100	0	0
RBM10	22	100	0	0	5	100	0	0	4	100	0	0
REL	22	100	0	0	5	100	0	0	4	100	0	0
RET	19	86	3	14	5	100	0	0	4	100	0	0
RICTOR	21	95	1	5	5	100	0	0	4	100	0	0
**RNF43**	22	100	0	0	5	100	0	0	4	100	0	0
**ROS1**	22	100	0	0	5	100	0	0	2	50	2	50
**RPTOR**	18	82	4	18	5	100	0	0	4	100	0	0
SDHA	21	95	1	5	5	100	0	0	4	100	0	0

SDHB	22	100	0	0	5	100	0	0	4	100	0	0
SETD2	18	82	4	18	5	100	0	0	4	100	0	0
SGK1	21	95	1	5	5	100	0	0	4	100	0	0
SMAD2	21	95	1	5	5	100	0	0	4	100	0	0
SMAD4	22	100	0	0	5	100	0	0	4	100	0	0
SMARCA4	22	100	0	0	4	80	1	20	3	75	1	25
SNCAIP	21	95	1	5	5	100	0	0	4	100	0	0
SOX2	22	100	0	0	5	100	0	0	3	75	1	25
SOX9	21	95	1	5	5	100	0	0	4	100	0	0
**SPEN**	17	77	5	23	5	100	0	0	3	75	1	25
STAG2	20	91	2	9	5	100	0	0	4	100	0	0
STAT3	21	95	1	5	5	100	0	0	4	100	0	0
STK11	22	100	0	0	5	100	0	0	4	100	0	0
SYK	22	100	0	0	5	100	0	0	4	100	0	0
TBX3	21	95	1	5	5	100	0	0	4	100	0	0
TEK	20	91	2	9	5	100	0	0	3	75	1	25
**TERT**	8	36	14	64	3	60	2	40	1	25	3	75
TET2	20	91	2	9	5	100	0	0	4	100	0	0
TGFBR2	22	100	0	0	5	100	0	0	3	75	1	25
**TP53**	14	64	8	36	1	20	4	80	2	50	2	50
**TSC1**	16	73	6	27	4	80	1	20	3	75	1	25
TSC2	22	100	0	0	5	100	0	0	3	75	1	25
**TYRO3**	21	95	1	5	4	80	1	20	4	100	0	0
U2AF1	20	91	2	9	5	100	0	0	4	100	0	0
VHL	21	95	1	5	5	100	0	0	4	100	0	0
WHSC1MMSET	22	100	0	0	5	100	0	0	4	100	0	0
ZNF703	20	91	2	9	4	80	1	20	4	100	0	0
